# Alpha-1-antichymotrypsin as a novel biomarker for diagnosis, prognosis, and therapy prediction in human diseases

**DOI:** 10.1186/s12935-022-02572-4

**Published:** 2022-04-19

**Authors:** Yanxia Jin, Weidong Wang, Qiyun Wang, Yueyang Zhang, Kashif Rafiq Zahid, Umar Raza, Yongsheng Gong

**Affiliations:** 1 Hubei Key Laboratory of Edible Wild Plants Conservation and Utilization, College of Life Sciences, Hubei Normal University, No. 11 Cihu Road, Huangshi District, Huangshi, 435002 China; 2 College of Life Sciences, Hubei Normal University, No. 11 Cihu Road, Huangshi District, Huangshi, 435002 China; 3 Suzhou Municipal Hospital, The Affiliated Suzhou Hospital of Nanjing Medical University, No.26 Daoqian Street, Suzhou, 215002 China; 4 Shenzhen Key Laboratory of Microbial Genetic Engineering, College of Life Science and Oceanography, Carson International Cancer Center, Shenzhen University, Shenzhen, Guangdong China; 5 Department of Biological Sciences, National University of Medical Sciences (NUMS), PWD Campus, Rawalpindi, Pakistan

**Keywords:** Alpha-1-antichymotrypsin, Inflammation, Cancer, Biomarker, Diagnosis, Prognosis, Therapeutic target

## Abstract

The glycoprotein alpha-1-antichymotrypsin (AACT), a serine protease inhibitor, is mainly synthesized in the liver and then secreted into the blood and is involved in the acute phase response, inflammation, and proteolysis. The dysregulation of AACT and its glycosylation levels are associated with tumor progression and recurrence, and could be used as a biomarker for tumor monitoring. In this review, we summarized the expression level, glycosylation modification, and biological characteristics of AACT during inflammation, neurodegenerative or other elderly diseases, and tumorigenesis, as well as, focused on the biological roles of AACT in cancer. The aberrant expression of AACT in cancer might be due to genetic alterations and/or immune by bioinformatics analysis. Moreover, AACT may serve as a diagnostic or prognostic biomarker or therapeutic target in tumors. Furthermore, we found that the expression of AACT was associated with the overall survival of patients with human cancers. Decreased AACT expression was associated with poor survival in patients with liver cancer, increased AACT expression was associated with shorter survival in patients with pancreatic cancer, and decreased AACT expression was associated with shorter survival in patients with early lung cancer. The review confirmed the key roles of AACT in tumorigenesis, suggesting that the glycoprotein AACT may serve as a biomarker for tumor diagnosis and prognosis, and could be a potential therapeutic target for human diseases.

**Supplementary Information:**

The online version contains supplementary material available at 10.1186/s12935-022-02572-4.

## Introduction

**Fig. 1:**
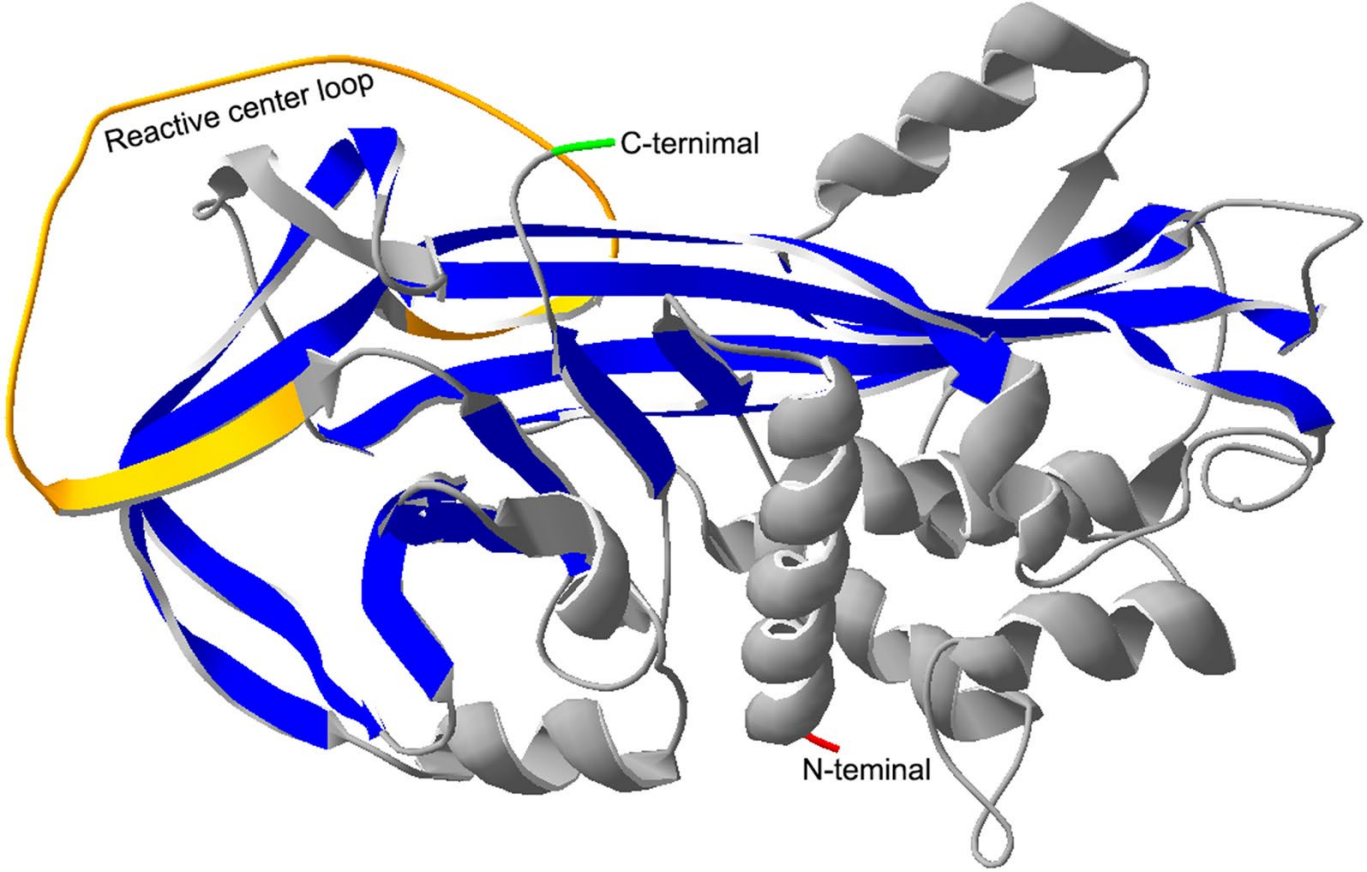
The 3D structure of AACT. The color red represents the N-terminal amino acid, green represents the C-terminal amino acid, yellow represents the reactive center loop, gray represents α-helixes and blue represents β-fold sheets. The amino acid sequences were derived from UniProt (https://www.uniprot.org/uniprot/P01011), and the 3D structure was constructed by the SWISS-MODEL WORKSPACE (http://swissmodel.expasy.org)

Alpha-1-antichymotrypsin (AACT), namely serpin family A member 3 (SERPINA3), is encoded by the serpin A3 gene and has 423 amino acids with a molecular weight of 47.651 kDa. We have remodeled the structure of AACT with the SWISS-MODEL WORKSPACE (http://swissmodel.expasy.org) [1–3] and SPDBV software (http://swissmodel.expasy.org/), which is based on Protein Data Bank files (PDB ID: 1qmn.1 A) and has 99.75% identity with 1qmn.1 A. The 3D structure of AACT is shown in Fig. 1. The protein structure of AACT consists of an α -helix, β -folded sheets and a reaction center loop (RCL) [4]. AACT is mainly synthesized in the liver and then secreted into the blood. Some reports have shown that AACT is also expressed in other organs, such as the brain and aorta [5, 6], and is also secreted in astrocytes [7]. As a serine protease inhibitor, AACT can inhibit neutrophil cathepsin G (CTSG) and mast cell chymase and protects cells or tissues from damage caused by proteolysis after inflammation [8], which is essential to maintain intracellular homeostasis and extracellular matrix reconstruction. The function of AACT is mainly involved in the acute phase response [9], inflammation [10], and proteolysis [11] and could be used as a biomarker for the diagnosis and prognosis of diseases [12], including liver cancer [13], pancreatic cancer [14, 15], lung cancer [16, 17], ovarian cancer [18], and diffuse large B-cell lymphoma [19].

Accumulating evidence has demonstrated a crucial role of protein glycosylation in biological processes, and abnormal glycosylation is tightly associated with tumorigenesis. The glycan sites, different types of glycosylation and complex sugar chain structure of AACT affect its structure and biological functions [20, 21] and are involved in the occurrence and development of diseases; for example, the changes in glycoform profiles in AACT reflected the physiological state of patients with sepsis [22]. Data from the UniProt and GeneCards databases show that AACT has six N-linked *N*-acetylglucosamine (GlcNAc) modification sites [23] (33, 93, 106, 127, 186 and 271), but their *N*-glycan structures are not clear. Santamaria et al. reported that the N-linked glycosylation modification of AACT in the nucleus has less complex form than secreted AACT, and perhaps the protease inhibitory activity of AACT helps some proteins escape the degradation of protease and transport into the nucleus [24].

However, the expression changes, glycan modification, and biological functions of AACT remain unclear during disease development. In our latest reports, we found that AACT is significantly decreased in the sera of patients with stage I NSCLC and reported that GlcNAc-modified AACT could be a novel biomarker for the early diagnosis of NSCLC [16, 17]. In this review, we have mainly focused on the differential expression and/or modifications of ACCT in disease to better understand its biological functions, especially clarifying the biological significance of AACT as a novel biomarker for diagnosis, prognosis, and therapy prediction in cancer.

## The biological significance of AACT in inflammatory diseases

AACT is also an acute-phase protein (APP) whose expression is changed during the inflammatory response and is involved in monitoring inflammatory diseases [25, 26]. In the cerebrospinal fluid (CSF) of patients with fibromyalgia, the expression of AACT is decreased and is involved in the inflammatory response and signal transduction [5]. Kroksveen et al. identified differentially expressed proteins using cerebrospinal fluid from the brain by iTRAQ labeling and proteomics and found that AACT could be used as a diagnostic or prognostic indicator for multiple sclerosis [27]. In heart tissue, the expression of AACT is directly regulated by miR-37, and elevated AACT and miR-37 play an important role in the pathology of patients with chronic heart failure (HF), thereby providing a potential therapeutic target [28]. Brioschi et al. reported that AACT with three other abundant plasma proteins in patients with HF, namely, neuropilin-2, beta 2 microglobulin, and complement component C9, could discriminate patients with HF from healthy subjects with sufficient accuracy [29] and could be used as a diagnostic biomarker. AACT is significantly increased in patients with aortic stenosis, which is important in the pathophysiology of this disease, and AACT may serve as a biomarker for the early diagnosis of aortic stenosis [6]. The level of AACT in serum is generally increased in patients with active tuberculosis [30]. Sun et al. screened new plasma markers to distinguish pulmonary tuberculosis from underlying infection with quantitative proteomics technology, and found that the combination of AACT, α-1-acid glycoprotein (alpha-1-acid glycoprotein 1, AGP1) and cadherin (E-cadherin, CDH1) could distinguish patients with pulmonary tuberculosis and underlying infection, with a sensitivity of 81.2% and specificity of 95.02% [26]. Therefore, AACT can be used as a biomarker for inflammatory diseases.

In addition, the glycosylation level of AACT is also changed in inflammatory diseases. For example, the glycosylation profile of AACT could be used as an indicator of physiological inflammatory status [31]. The glycan structure of the APP is dynamically modified by circulating glycosidase or glycosyltransferase in the inflammatory environment. AACT is highly glycosylated in acute inflammatory reactions, and the glycan form of fucosylated modification of SLe^x^ was significantly increased [32, 33]. In septic episodes, the alteration of AACT glycoforms such as increased fucosylation and additional branching of *N*-glycans could be used to monitor sepsis progression, and the individual AACT glycoproteoform profiles are unique, which may be linked with physiological states [22].

## The role of AACT in neurodegenerative or related elderly diseases

**Fig. 2:**
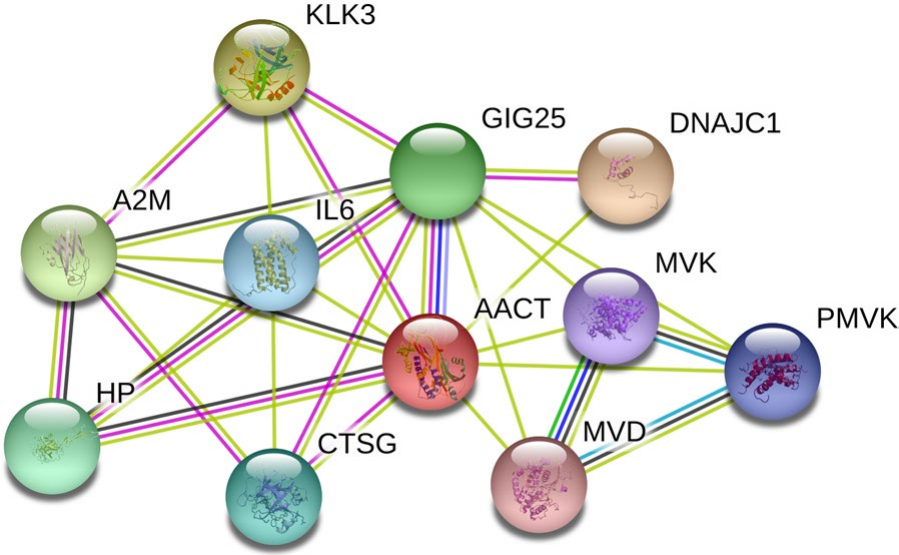
The protein-protein interaction with AACT. The data were analyzed by STRING (https://string-db.org). DNAJC1: DnaJ homolog subfamily C member 1; KLK3: prostate-specific antigen; A2M: Alpha-2-macroglobulin; GIG25: Serpin peptidase inhibitor; HP: Haptoglobin; CTSG: Cathepsin G; IL6: Interleukin-6; PMVK: Phosphomevalonate kinase; MVK: Mevalonate kinase; MVD: Diphosphomevalonate decarboxylase

AACT promotes the secretion of cytokines, including IL-6 and tumor necrosis factor-alpha (TNF-α), by regulating the NF-κB signaling pathway to be involved in nervous system diseases [34]. We analyzed the proteins that interacted with AACT by STRING, which found that AACT interacted with IL-6 (Fig. 2). AACT is a major component of nerve fiber nodules, which play an important role in the pathogenesis of Alzheimer’s disease (AD). The protein expression of AACT is higher in the CSF and serum of patients with AD [5], Aβ amyloid plaque deposits with increasing AACT concentration, and AACT can activate c-Jun N-terminal kinase, further mediating the hyperphosphorylation of tau protein and leading to neurofibrillary tangles [35]. Braghin et al. found that AACT induced mRNA expression and released TNF-α in murine microglial cells, promoting NF-κB translocation into the nucleus in AD pathogenesis, which suggested that inhibition of AACT could be useful for the treatment of AD [36]. Moreover, Chen et al. reported that the N-linked glycosylation pattern of alpha-1-antichymotrypsin was altered in AD, which could contribute to elucidating the role of glycosylation in AD progression [37]. Lanni et al. found that the sialic acid levels of AACT were significantly reduced in AD [38]. For other diseases of aging, Liu et al. reported that AACT may serve as a biomarker for diagnosing amnestic mild cognitive impairment (aMCI) [39]. AACT combined with other proteins can be used as predictor of the five-year risk of death in older men through high-throughput serum proteomics identification [40].

## The biological significance of AACT in human cancer

### The abnormal expression and glycosylation level of AACT in tumors

AACT exhibits low specificity in cancers, and the protein or glycosylation level of AACT is aberrantly expressed in various tumors, and associated with survival and tumor progression, including ovarian cancer [18], colorectal cancer [41], melanoma [42], and endometrial cancer [43], as well as can be used as a biomarker for cancer monitoring. Weiz et al. reported that biantennary and tetraantennary glycoforms containing Lewis^x^ structures in AACT were increased in epithelial ovarian cancer, while the β (1–4) branch and triantennary-branched structures were reduced [18]. The glycoforms containing SLe^x^ of AACT and other APPs, such as AGP and haptoglobin, have been elevated in ovarian cancer [44], and may be candidates for monitoring breast cancer progression [45]. In prostate cancer, prostatic specific antigen (PSA) is a useful prostate cancer marker that is complexed with AACT, as shown in Fig. 2, and the PSA-AACT levels were increased in moderately differentiated prostate cancer tissues [46]. Julia et al. also found that the combination of AACT and PSA significantly increased the diagnostic rate of prostate cancer compared to PSA alone, with an AUC value of 0.71 [47].

**Fig. 3:**
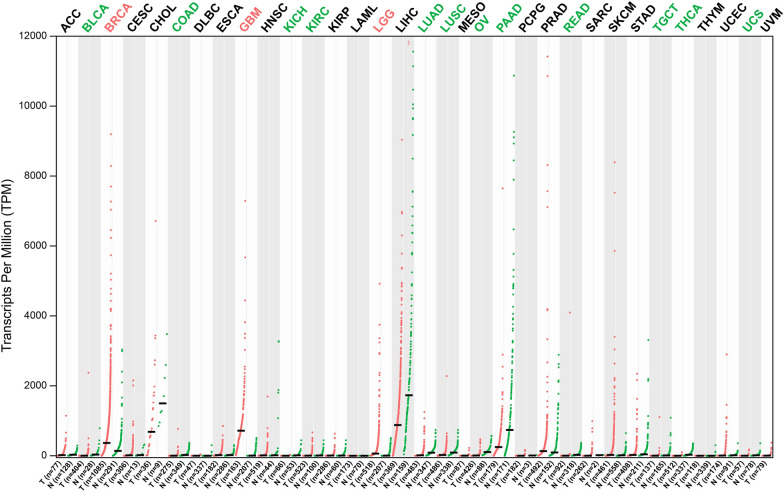
The gene expression profile across all tumor samples (T) and paired normal tissues (N) (dot plot). Each dot represents the AACT mRNA expression level of the samples. Data were downloaded from the GEPIA database (http://gepia.cancer-pku.cn/detail.php?gene=SERPINA3). ACC: Adrenocortical carcinoma; BLCA: Bladder urothelial carcinoma; BRCA: Breast invasive carcinoma; CESC: Cervical squamous cell carcinoma and endocervical adenocarcinoma; CHOL: Cholangio carcinoma; COAD: Colon adenocarcinoma; DLBC: Lymphoid neoplasm diffuse large B-cell lymphoma; ESCA: Esophageal carcinoma; GBM: Glioblastoma multiforme; HNSC: Head and neck squamous cell carcinoma; KICH: Kidney chromophobe; KIRC: Kidney renal clear cell carcinoma; KIRP: Kidney renal papillary cell carcinoma; LAML: Acute myeloid leukemia; LGG: Brain lower grade glioma; LIHC: Liver hepatocellular carcinoma; LUAD: Lung adenocarcinoma; LUSC: Lung squamous cell carcinoma; MESO: Mesothelioma; OV: Ovarian serous cystadenocarcinoma; PAAD: Pancreatic adenocarcinoma; PCPG: Pheochromocytoma and paraganglioma; PRAD: Prostate adenocarcinoma; READ: Rectum adenocarcinoma; SARC: Sarcoma; SKCM: Skin cutaneous melanoma; STAD: Stomach adenocarcinoma; TGCT: Testicular germ cell tumors; THCA: Thyroid carcinoma; THYM: Thymoma; UCEC: Uterine corpus endometrial carcinoma; UCS: Uterine carcinosarcoma; UVM: Uveal melanoma

We also used the GEPIA database to test the mRNA expression levels of AACT in different tumor tissues and found that the expression of AACT was significantly differentially expressed in multiple cancer types compared to their normal counterparts (Fig. 3). These cancer types included invasive breast carcinoma (BRCA), glioblastoma multiforme (GBM), lung adenocarcinoma (LUAD), lung squamous cell carcinoma (LUSC), ovarian serous cystadenocarcinoma (OV), pancreatic adenocarcinoma (PAAD), etc.

### Why is AACT abnormally expressed in cancer?

**Fig. 4:**
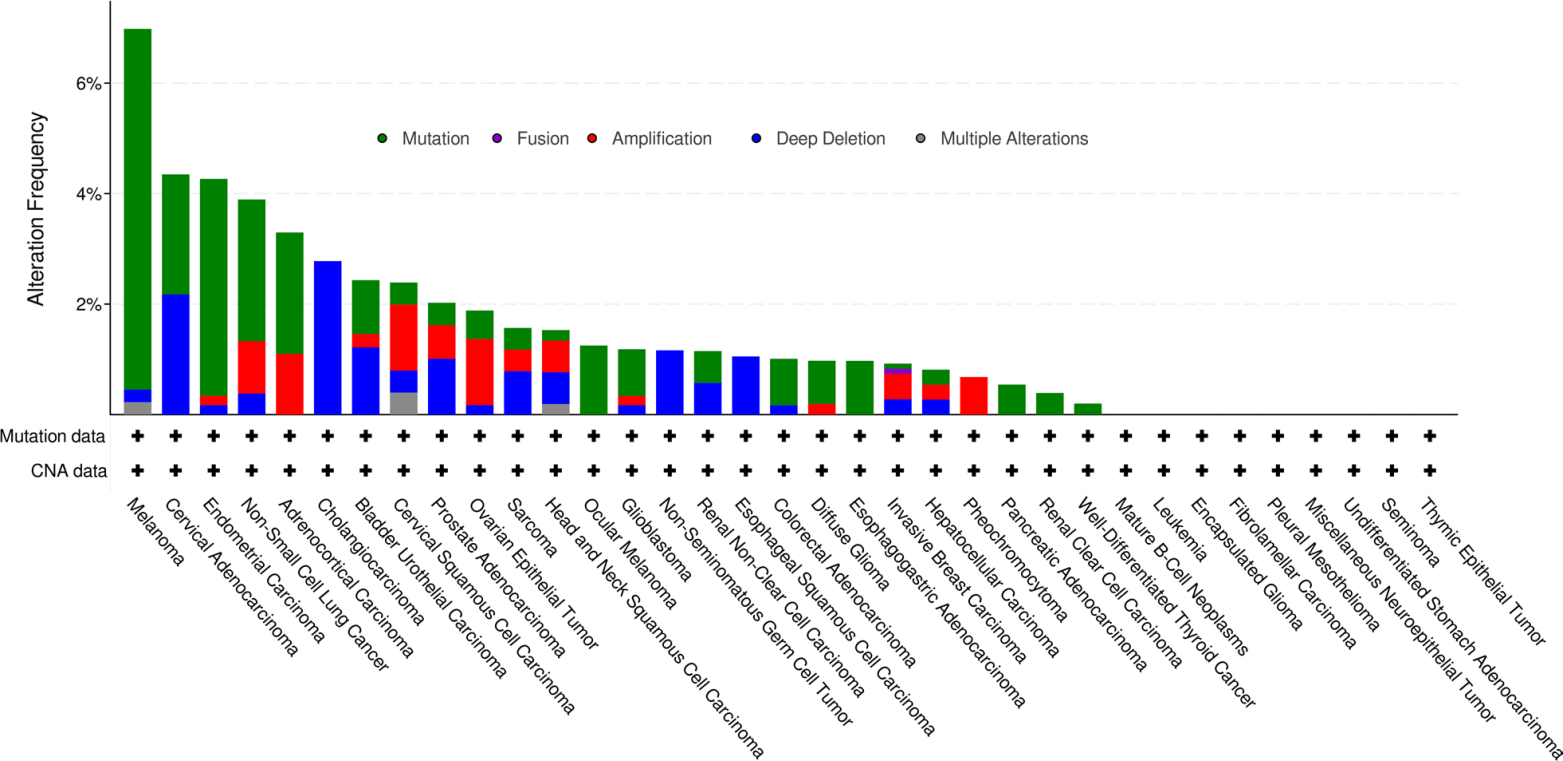
The gene alterations of AACT in tumors. Summary of the gene alterations for AACT in various cancers. Data were downloaded from cBioPortal (http://www.cbioportal.org/)

Aberrant AACT expression might be induced through gene mutation, deep deletion, fusion, amplification or multiple alterations in tumors. We performed a statistical analysis of the DNA alteration frequency of AACT in various cancer types by using the cBioPortal database (http://www.cbioportal.org/) (Fig. 4) [48]. Interestingly, we recorded 6.94%, 3.89%, and 0.81% genetic alteration frequencies of AACT in 444 melanoma cases, 1053 NSCLC cases, and 389 liver cancer cases, respectively. The changes in the expression levels of AACT may be regulated by different signaling pathways or immune microenvironments, such as immunosuppression in glioma [49, 50].

Furthermore, we also used the COSMIC database to analyze the mutation distribution of AACT in different tissues (Table 1), which indicated that the mutation rate of AACT was different in tissues and diseases. For example, 3.48% of cases were found to exhibit point mutations in lung adenocarcinoma, and in bladder carcinoma, the point mutation, copy number variation, and hypomethylation were 1.44%, 0.5% and 10.62% of the cases, respectively.

**Table 1:** The distribution of mutations across the primary tissue types that are curated by COSMIC database

Tissue/sub-tissue	Histology	Sub histology	Point mutations (%)	Copy number variation (%)		Hypomethylation (%)
				Gain	Loss	
Bile duct/gallbladder	Carcinoma	Small cell carcinoma	5.88 (n = 17)	/	/	/
Bile duct/bile duct	Carcinoma	Adenocarcinoma	3.5 (n = 457)	/	/	/
Breast	Carcinoma	/	0.14 (n = 1429)	0.18 (n = 1131)	0.35 (n = 1131)	/
Central nervous system/cerebellum	Glioma	Astrocytoma Grade IV	7.14 (n = 14)	/	/	/
Cervix	Carcinoma	Squamous cell carcinoma	2.83 (n = 318)	/	0.33 (n = 299)	/
Endometrium	Carcinoma	Endometrioid carcinoma	3.61 (n = 581)	/	0.19 (n = 530)	/
Haematopoietic and lymphoid	Lymphoid neoplasm	Follicular lymphoma	2.33 (n = 43)	/	/	/
Kidney	Carcinoma	Papillary renal cell carcinoma	0.59 (n = 340)	/	1.05 (n = 286)	/
Liver	Carcinoma	Hepatocellular carcinoma	0.69 (n = 1012)	0.15 (n = 660)	0.15 (n = 660)	/
Lung	Carcinoma	Adenocarcinoma	3.48 (n = 1035)	0.53 (n = 375)	0.27 (n = 375)	/
Lung	Carcinoma	Squamous cell carcinoma	2.62 (n = 762)	0.4 (n = 500)	/	/
Ovary	Carcinoma	Serous carcinoma	0.59 (n = 673)	0.18 (n = 568)	0.18 (n = 568)	/
Oesophagus	Carcinoma	Adenocarcinoma	0.64 (n = 467)			
Pancreas	Carcinoma	Ductal carcinoma	0.7 (n = 1281)			
Prostate	Carcinoma	Adenocarcinoma	0.48 (n = 1462)	/	/	/
Skin	Malignant melanoma	/	8.35 (n = 826)			
Stomach	Carcinoma	Adenocarcinoma	2.35 (n = 596)	/	/	/
Thyroid	/	neoplasm	6.67 (n = 15)	/	/	/
Upper aerodigestive tract/ head neck	Carcinoma	Squamous cell carcinoma	0.8 (n = 519)	0.58 (n = 626)	/	0.6 (n = 496)
Urinary tract/Bladder	Carcinoma	/	1.44 (n = 555)	0.5 (n = 399)	/	10.62 (n = 273)

The data was derived from the COSMIC database (https://cancer.sanger.ac.uk/cosmic/gene/analysis?ln=SERPINA3_ENST00000393078), and there are 40,656 unique samples were used in this database, of which 506 unique samples with mutations were distributed in 40 types of tissues. n represents the number of samples

**Fig. 5:**
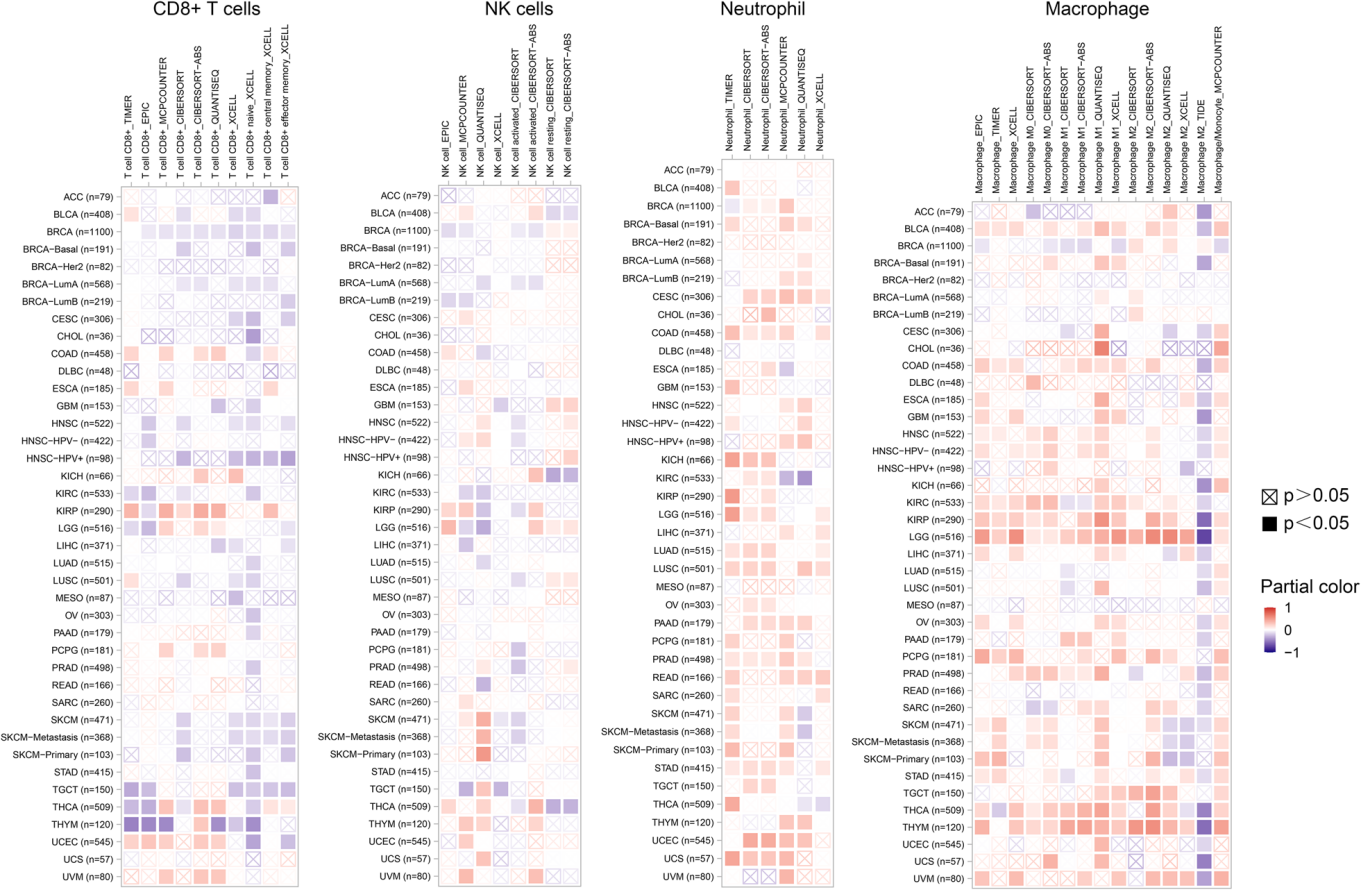
The correlation between AACT expression and immune infiltration in tumors. The correlation between AACT expression and immune infiltration in 40 types of tumors was analyzed by Timer 2.0 software (http://timer.cistrome.org/). The NK cells, neutrophils, macrophages and CD8 + T cells were selected to analyze the correlation with AACT expression in tumors by Spearman’s test. Red represents a significantly positive correlation (p < 0.05), and blue represents a significantly negative correlation (p < 0.05). The depth of color represents the value of the correlation coefficient

In addition, the abnormal expression of AACT in cancer may be associated with immunity. The immune microenvironment and immune cells are closely related to cancer occurrence, and most tumor-infiltrating immune cells are imbalanced in the development of tumors [51]. We analyzed the correlation between AACT expression and immune cells, including NK cells, CD8+ T cells, macrophages and neutrophils, in 40 types of tumors by Timer 2.0 software. Different immune invasion assessment algorithms have different results, such as the ESTIMATE algorithm [52], XCELL (http://xcell.ucsf.edu/), MCP-counter (http://github.com/ebecht/MCPcounter), and CIBERSORT (https://cibersort.stanford.edu/index.php), and the results are shown in Fig. 5. For example, in cervical squamous cell carcinoma (CESC), AACT has a positive correlation with neutrophils and a negative correlation with CD8+ T cells with the XCELL algorithm. However, the results showed that AACT had no significant correlation with CD8+ T cells and a positive correlation with NK cells, macrophages and neutrophils with the MCP-counter algorithm. However, the detailed relationship between AACT expression and immunity in tumors needs to be further elucidated by experiments.

As above, it may be the reason to clarify the abnormal expression of AACT in cancer. However, the molecular mechanisms of AACT dysregulation in every tumor still need to be investigated further.

### The biological roles of AACT in cancer

#### AACT as a prognostic biomarker or therapeutic target in glioma

AACT could be a candidate biomarker for the early diagnosis of glioblastoma, which was identified using quantitative proteomics detection [53]. Furthermore, Lara-Velazquez et al. reported that AACT overexpression led to an increase in cell migration, while silencing of AACT decreased cell migration in glioblastoma and increased the survival of mice, which is positively correlated with glioma grade and negatively correlated with the prognosis of patients with GBM [54, 55]. AACT expression regulates the MAPK signaling pathway and matrix metalloproteinase expression, and AACT may serve as a potential immunomodulator and therapeutic target in GBM [49]. Yuan et al. found that AACT expression was involved in immune suppression in glioma, and higher AACT expression was associated with lower CD4^+^ T cell levels, which indicated that AACT played a key role in the occurrence of glioma [50].

#### AACT as a diagnostic biomarker or therapeutic target in hepatocellular carcinoma

Alterations in core fucosylation, increased sialylation and glycan branching have been identified in the serum of patients with hepatocellular carcinoma (HCC), and are involved in tumor development [56]. Both the expression levels of AACT and its glycosylation levels were abnormal in the sera of patients with HCC [57]. Ji et al. applied hydrophilic interaction liquid chromatography (HILIC) to enrich N-linked glycopeptides from plasma in patients with liver cancer and then analyzed them by nano-LC/ESI-MS/MS. The N-linked glycopeptide modification sites N106 and N271 of AACT were detected by collision-induced dissociation (CID) and higher energy C trap dissociation (HCD) mass spectrometry, and further identified that the fucosylation modification of N-linked glycopeptides in AACT was mainly tri- and/or tetra-antennary glycan structure [58]. The *aleuria aurantia* lectin (AAL) was used for enriching the plasma from liver cancer and combined with multiple reaction monitoring (MRM) quantitative proteomic analysis, which found that AACT captured by AAL could significantly distinguish liver cancer from healthy samples, with an AUC of 0.927 and 90.0% sensitivity at a specificity of 83.3%, which suggested that AACT could be a potent biomarker for patients with HCC [13].

**Fig. 6:**
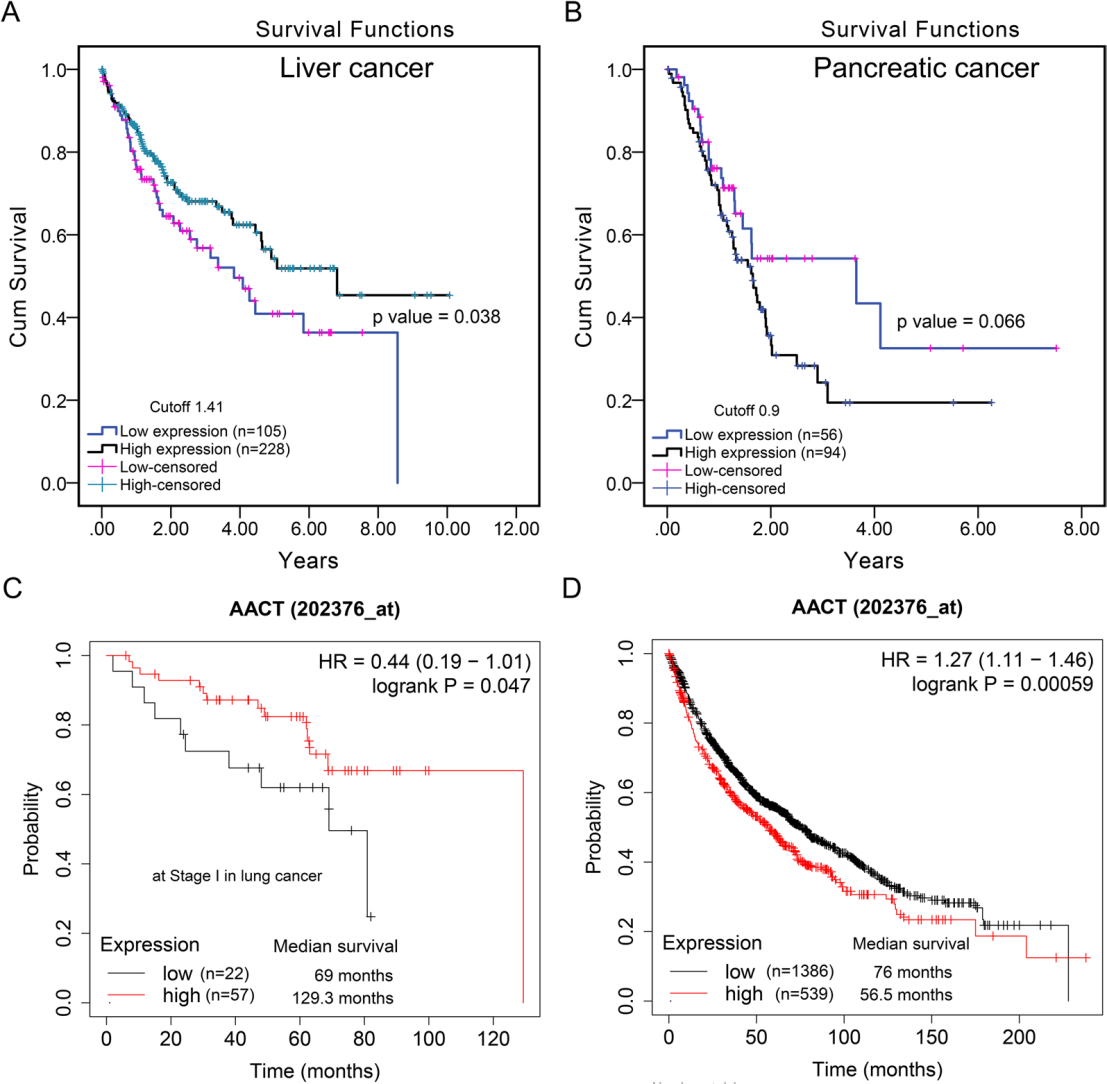
Analysis of the association between mRNA expression level of AACT and overall survival in tumors. **A**, **B** The fragment per kilobase of exon model per million mapped reads (FPKM) data of AACT mRNA expression levels were downloaded from the Human Protein Atlas database (https://www.proteinatlas.org/ENSG00000196136-SERPINA3) (Additional file 1), and overall survival analysis was performed using the Kaplan–Meier method, followed by the log-rank test with SPSS 24.0 software. The FPKM cutoff of AACT mRNA expression in liver cancer was 1.41 (**A**) and in pancreatic cancer was 0.9 (**B**). **C**, **D** The overall survival analysis was performed using the Kaplan–Meier Plotter database (http://www.kmplot.com/analysis/index.php?p=service&cancer=lung). The parameter settings: Affy ID: 232376_at; Split patients by: Auto select best cutoff; Survival: OS; History: all; grade: all; Gender: all; Smoking history: all; Cox regression: univariate. **C**. In early-stage lung cancer. Stage: 1; AJCC stage T: 1; AJCC Stage N: 0; AJCC stage M: 0. **D**. In lung cancer. Stage: all; AJCC stage T: all; AJCC Stage N: all; AJCC stage M: all

Santamaria et al. reported that the protein level of AACT is normally expressed in healthy noncancerous liver tissue and decreased in tissues and cells of liver cancer. The decreased expression of AACT was associated with liver cancer development, while AACT overexpression inhibited the proliferation of liver cancer cells. In vitro studies indicated that AACT inhibited DNA synthesis by inhibiting DNA polymerase activity, and found that AACT was located in the nucleus through binding tightly with chromatin, which promoted chromatin to be in a condensed state and prevented cell proliferation [24]. Zhu et al. found that AACT acts as a tumor suppressor in liver cancer and inhibits the PI3K/AKT/mTOR pathway by activating PTEN, thus inhibiting the development and metastasis of liver cancer. We analyzed the mRNA expression levels of AACT and found that AACT expression was associated with the overall survival of liver cancer in 333 patients, and decreased expression of AACT was associated with poorer survival of patients with liver cancer (Fig. 6A). These reports suggest that AACT can also be used as a target for treatment intervention in liver cancer [59].

#### AACT as a diagnostic or prognostic biomarker in pancreatic cancer

Pancreatic cancer is an intractable malignancy with a 5-year survival rate lower than 5% [60]. AACT could be used as a biomarker for the diagnosis of pancreatic cancer, and the combination of AACT, thrombospondin-1 (THBS1) and peptides containing single amino acid variants (SAAVs) has shown an improved diagnostic performance in the identification of pancreatic cancer from healthy controls, with an AUC of 0.98, which was demonstrated to improve the survival rate if diagnosed at an early stage [15]. Moreover, Nie et al. also used AAL to extract serum fucosylated glycoproteins with quantitative proteomics, and found that the combination of AACT, THBS1, and haptoglobin (HPT) could differentiate patients with pancreatic cancer from healthy controls, with an AUC of 0.95 [14]. AACT, vimentin, β-catenin, and α-1 antitrypsin can be used as new markers for the diagnosis of solid serous cystadenoma (SCA) of the pancreas, which is a rare type of pancreatic solid tumor [61]. AACT and other markers were used to identify extraordinarily rare pancreatic-type acinar cell carcinoma in the stomach [62].

In addition, Roberts et al. showed that AACT was negatively correlated with the overall survival of patients with pancreatic cancer, and increased AACT expression was associated with shorter survival of patients with pancreatic cancer [63]. The obtained results in this review are in line with previously published data, as shown in Fig. 6B. Moreover, AACT was used for survival prediction of patients with pancreatic cancer following gemcitabine treatment [64]. Together, these data suggest that AACT can be an effective prognostic indicator for advanced pancreatic cancer. However, the mechanism and related functions of AACT in pancreatic cancer still need to be investigated further.

Aberrant glycosylation has been identified in pancreatic cancer [65, 66], and glycosciences.de software (http://www.glycosciences.de/) was used to mine the glycoforms, such as fucosylation, sialyl Lewis A and Lewis^x^, for the early detection of pancreatic cancer [67–69]. *N*-glycosylation has a large variation in the metastatic pathogenesis of pancreatic cancer [70]. Currently, the glycosylation of AACT in pancreatic cancer has not been reported.

#### AACT as a diagnostic or prognostic biomarker in lung cancer

Previous studies have reported that AACT is also synthesized in lung adenocarcinoma cells, and the expression levels of AACT in lung cancer cell lines are associated with tumor progression, especially tumor growth. Zhang et al. reported that AACT in urine can be used as a biomarker for the diagnosis of NSCLC, and the expression level of AACT in the urine of patients with stage IV NSCLC was increased. The expression level of AACT is increased in advanced lung cancer tissues, and the level of AACT was found to be significantly increased in the plasma of patients with metastatic lung cancer [71]. In our previous study, we found that the expression level of the glycoprotein AACT and its GlcNAcylation level were decreased in the serum of patients with early-stage NSCLC, and were especially significantly decreased in stage IA, but AACT and its GlcNAcylation levels were restored and presented an increasing trend in late-stage NSCLC [16]. Perhaps the role of AACT in tumorigenesis and progression depends not only on its expression level but also on the complex regulation of its glycan modification levels. A study reported that the expression level of beta 1,6-N-acetylglucosaminyl transferase (GnT-V) was decreased in 50% of patients with NSCLC, resulting in a decrease in the βl,6 GlcNAc branch of the *N*-glycan structure, which was associated with a poor prognosis of NSCLC [72]. The decrease in the GlcNAcylation level of AACT in the early occurrence of NSCLC may be related to the downregulated expression of GnT-V. The glycosylation level changes of AACT may be associated with glycosyltransferase, but there are no directly related reports. To date, there are no related reports about the function of AACT in lung cancer.

Our previous studies showed that GlcNAcylated AACT could effectively distinguish stage I from benign samples by lectin-ELISA tests, with an AUC value of 0.932 and 90.9% sensitivity at a specificity of 93.8%. Moreover, GlcNAcylated AACT could also effectively distinguish stage I from tumor-free (healthy and benign) samples, with an AUC value of 0.908 and 90.9% sensitivity at a specificity of 86.2%. Combining the GlcNAcylated AACT and CEA significantly improved the specificity of CEA in the diagnosis of early-stage NSCLC [16]. Furthermore, we also found that combining GlcNAcylated AACT and GlcNAcylated serum paraoxonase/arylesterase l (PONl) can provide better accuracy in distinguishing early NSCLC from tumor-free samples, and the AUC for this combination was as high as 0.940, with 94.4% sensitivity and 90.2% specificity [17]. These results show that GlcNAc modified AACT with high sensitivity and specificity can be used as a novel biomarker for the early diagnosis of NSCLC.

In addition, the correlation between AACT mRNA expression levels and the overall survival of lung cancer was also analyzed by the Kaplan Meier Plotter database. In early-stage lung cancer, the median survival was 69 months, with lower expression of AACT in patients with early lung cancer patients (n = 22) and with shorter survival; the median survival was 129.3 months, with higher expression of AACT in patients with early lung cancer (n = 57) with longer survival (Fig. 6C), which suggested that mRNA expression of AACT was strongly associated with the survival of patients. However, in all patients with lung cancer, the median survival was 76 months, with lower expression of AACT in patients with lung cancer (n = 1386) and with longer survival. The median survival was 56.5 months, with a higher expression of AACT in patients with lung cancer (n = 539) (Fig. 6D), which suggested that AACT could be used as a prognostic marker for therapeutic efficiency and survival evaluation in lung cancer.

## Conclusion and overall perspectives

Here, we have provided the updated structure, glycosylation modification, and biological characteristics of AACT during inflammatory and neurodegenerative diseases and tumorigenesis (Table 2). As a serine protease inhibitor and an APP, the protein expression and glycan modification of AACT, such as fucose, sialyl acid, SLe^x^, GlcNAc, etc., is changed in inflammatory conditions and cancers. In addition, we have described the correlation between AACT expression and disease progression, particularly in cancer. The correlation between AACT expression and prognosis in inflammatory or neurodegenerative diseases is different from that found in tumors, which is most likely due to the different regulation of the immune microenvironment, but the underlying molecular mechanism still needs to be explored further. AACT expression is associated with the overall survival of cancer patients; however, the occurrence mechanisms of AACT in other diseases are still unknown and inhibitors that regulate AACT levels have not been reported, so further research on AACT-mediated disease progression and related therapy is still needed. This review provides recent findings that may be useful for designing basic research in investigating glycoprotein AACT as diagnostic and prognostic biomarkers and therapeutic targets in tumors.

**Table 2:** List the expression changes and roles of alpha-1-antichymotrypsin in human diseases

Diseases		AACT derived	Abnormal expression of AACT	Roles	Reference
Inflammatory diseases	Fibromyalgia	CSF	↓	Involve in the inflammatory response and signal transduction	[5]
	Multiple sclerosis	CSF	↑	Diagnostic or prognostic indicator	[27]
	Heart failure	Plasma	↑	Therapeutic or diagnostic target	[28, 29]
	Aortic stenosis	Plasma	↑	Indicator for early diagnosis	[6]
	Active tuberculosis	Serum	↑	Use for diagnosis	[30]
Neurodegenerative diseases	AD	Serum, CSF	↑	Lead to neurofibrillary tangles in AD	[5, 35]
	aMCI	Serum, CSF	↑	Biomarker for diagnosing aMCI	[39]
Cancer	Ovarian cancer	Serum, ascites	↑	Biomarker for diagnosis	[18, 73]
	Prostate cancer	Tissues	↑	Increased the rate of prostate diagnosis	[46, 47]
	glioblastoma	CSF	↑	Therapeutic target in GBM	[49]
	HCC	Tissues	↓	Tumor suppressor and as a target for treatment intervention	[24, 59]
	Pancreatic cancer	Serum	↑	Diagnostic or prognostic indicator	[14, 64]
		Tissues	↑		[61–63]
	Lung cancer	Serum	in early-stage:↓; in late-stage:↑	Early diagnostic biomarker of NSCLC	[16, 17]
		Urine	in late-stage: ↑	Diagnostic biomarker of NSCLC	[71]
	Colorectal cancer	Tissues	↓	Potential biomarker for CRC progression	[74]

CSF: cerebrospinal fluid; AD: Alzheimer’s disease syndrome; aMCI: amnestic mild cognitive impairment; GBM: glioblastoma multiforme; HCC: hepatocellular carcinoma; NSCLC: non-small cell lung cancer; CRC: colorectal cancer; ↓: down-regulated. ↑: up-regulated

## Supplementary Information

**Additional file 1: Table S1. **List the samples in liver cancer and pancreatic cancer used in this manuscript.

## Data Availability

All data generated or analyzed during this study are included in this published article and its supplementary information files.

## Abbreviations

AACT: Alpha-1-antichymotrypsin
CTSG: Neutrophil cathepsin G
RCL: Reactive center loop
GlcNAc: *N*-acetylglucosamine
APP: Acute-phase protein
HF: Heart failure
AGP1: Alpha-1-acid glycoprotein 1
CDH1: E-cadherin
TNF-α: Tumor necrosis factor
PSA: Prostatic specific antigen
HILIC: Hydrophilic interaction liquid chromatography
CID: Collision-induced dissociation
HCD: Higher energy C trap dissociation
AAL: *Aleuria aurantia* Lectin
MRM: Multiple reaction monitoring
THBS1: Thrombospondin-1
SAAVs: Single amino acid variants
HPT: Haptoglobin
SCA: Solid serous cystadenoma
GnT-V: Beta 1,6-*N*-acetylglucosaminyl transferase
ACC: Adrenocortical carcinoma
BLCA: Bladder urothelial carcinoma
BRCA: Breast invasive carcinoma
CESC: Cervical squamous cell carcinoma and endocervical adenocarcinoma
CHOL: Cholangio carcinoma
COAD: Colon adenocarcinoma
DLBC: Lymphoid neoplasm diffuse large B-cell lymphoma
ESCA: Esophageal carcinoma
GBM: Glioblastoma multiforme
HNSC: Head and neck squamous cell carcinoma
KICH: Kidney chromophobe
KIRC: Kidney renal clear cell carcinoma
KIRP: Kidney renal papillary cell carcinoma
LAML: Acute myeloid leukemia
LGG: Brain lower grade glioma
LIHC: Liver hepatocellular carcinoma
LUAD: Lung adenocarcinoma
LUSC: Lung squamous cell carcinoma
MESO: Mesothelioma
OV: Ovarian serous cystadenocarcinoma
PAAD: Pancreatic adenocarcinoma
PCPG: Pheochromocytoma and paraganglioma
PRAD: Prostate adenocarcinoma
READ: Rectum adenocarcinoma
SARC: Sarcoma
SKCM: Skin cutaneous melanoma
STAD: Stomach adenocarcinoma
TGCT: Testicular germ cell tumors
THCA: Thyroid carcinoma
THYM: Thymoma
UCEC: Uterine corpus endometrial carcinoma
UCS: Uterine carcinosarcoma
UVM: Uveal melanoma
CSF: Cerebrospinal fluid
AD: Alzheimer's disease syndrome
aMCI: Amnestic mild cognitive impairment
GBM: Glioblastoma multiforme
HCC: Hepatocellular carcinoma
NSCLC: Non-small cell lung cancer
CRC: Colorectal cancer
